# “Emotional regulation” or “affective regulation”?

**DOI:** 10.3389/fpsyg.2024.1429361

**Published:** 2024-11-07

**Authors:** Ruth Mª de Jesús Gómez, María A. Cornu-Labat

**Affiliations:** ^1^Faculty of Education and Psychology, Universidad Francisco de Vitoria, Madrid, Spain; ^2^Psychology Department, Universidad de Navarra, Pamplona, Spain

**Keywords:** emotional regulation, affective regulation, emotional intelligence, emotional competence, affectivity

## Abstract

In recent decades, there has been an increased interest in psychology to understand the emotional experience. This growing interest has led to a proliferation of terms, among which regulation, intelligence, and emotional competence stand out. Research in these areas has facilitated a better understanding of what emotion entails and how to intervene in it. However, this study highlights that these contributions are insufficient if one aims to understand and intervene in how reality affects each person. In this sense, there is an advocacy for the recovery of the term affectivity, as it addresses all affective experiences and, therefore, is broader and more integrative.

## 1 Introduction

For more than two decades, there has been increasing interest in psychology in understanding individuals' emotional experiences. Currently, there is awareness of the importance emotions play in the development of a balanced personality, making it one of the main areas of research. This has led to a proliferation of constructs and corresponding research aimed at explaining the emotional phenomenon and how to intervene in it (Duckworth et al., 2009; Gross, 2014).

The concept of emotional regulation is one of the most prominent in research (Adrian et al., 2011), as it addresses the fundamental concern: “How can I manage my emotions?” This question is shared with constructs such as emotional intelligence and emotional competence.

Any of the aforementioned terms exclusively address emotional experience without taking into account other sensitive experiences such as sentiment, affection, or mood.

Although there is no unanimity in how to define what an emotion is, there is some agreement on what it involves: the perception of a sensation, a physiological change, and a mobilization toward action. Compared to other affective experiences, emotion is considered the most reactive, immediate, brief, and intense (Reeve, 2009). While feelings involve greater cognitive engagement and durability, with less intensity. Emotion differs from mood in that while emotion is a reaction to a real or imaginary event, which is more or less significant and concrete, the origin of mood is more diffuse and undefined (Goldsmith, 1994). The influence of emotion is primarily on behavior, whereas mood affects cognition and attitude (Davidson, 1994). Moods last longer than emotions (Ekman, 1994). Affections are our preferences, the things and people we care about.

We can find in various publications (Hervás and Vázquez, 2006) the distinction between emotion and affectivity, for example, when discussing regulation. As will be shown later, we can speak of emotional regulation when referring to intervening in what emotions we feel and with what intensity, and of affective regulation when what is regulated is mood. However, the use of the term affective or affectivity is scarce. We do not find in any of the developments of regulation, competence, or emotional intelligence, what relationship emotional experience has with affect and mood. From these proposals, emotion is approached as an isolated phenomenon, not in relation to other sensitive experiences. Is it possible to fully understand this phenomenon without putting it into relation with affect and mood? By conceptualizing affectivity as the capacity to be impacted by reality (Martínez, 2018), its indispensable role in forming a healthy personality and achieving a fulfilling life is confirmed. To achieve proper personality development, it is necessary to consider the person as a whole, contemplating all dimensions, with special attention to their affective dimension.

Currently, we encounter a terminological dilemma that affects the understanding and approach to the affective dimension. Although the most commonly used term is “emotion,” it seems to refer only to one of the various affective experiences of the human being. However, in textbooks on the psychology of emotion, other experiences related to it are taken into account, such as mood, affects, or feelings.

With all of this in mind, we are faced with the following question: what term should we use to refer to the impact of life events on the individual and their subsequent sensitive experience? In this article, it will be argued that the term “emotional regulation,” along with its counterparts “emotional intelligence” and “emotional competence,” while making valuable contributions to this issue, are not sufficient because they do not take into account the affective dimension of the human being and its nature. We will advocate for the recovery of the term “affectivity,” which allows for the inclusion of isolated phenomena and understanding their integration into the wholeness of the individual, making it particularly interesting for clinical and/or educational intervention.

To this end, in the first part of the work, we will analyze the inadequacy of the construct of emotional regulation and similar terms such as emotional intelligence and emotional competence to address the objective of integrating our emotional experiences. The next question we pose is the appropriateness of reclaiming the term “affectivity.”

## 2 Emotional regulation

Emotional regulation refers to “those processes by which individuals exert influence over the emotions they have, when they have them, and how they experience and express them” (Gross, 1999, p. 275). These processes can be conscious or unconscious, with controlled regulation typically involving more effort than more automatic regulation (Gross and Thompson, 2007). They are aimed at intervening primarily in the intensity and duration of emotional experience (Thompson, 1994). It is difficult to differentiate emotionality from its regulation (Cole et al., 2004).

It's a complex term because it encompasses diverse experiences. Both pleasant and unpleasant emotions are regulated (Hervás and Vázquez, 2006). Both beneficial processes, such as emotional identification and expression, and harmful ones, such as drug use, can be employed. Therefore, a distinction can be made between adaptive and maladaptive regulation processes (Hervás and Vázquez, 2006). There is also talk of dysregulation, understood as the absence or delay in employing regulation strategies or their ineffectiveness (Hervás and Vázquez, 2006). It's important to consider that these processes can be both intrinsic and extrinsic (Thompson, 1994; Eisenberg et al., 2010), implying a vast field of research with strong educational implications when addressing the role of others in the personal development of emotional regulation processes.

Authors like Thompson (1994) include in their definition of emotional regulation the fact that we regulate to achieve our own goals. Among the authors who have addressed the question of what we seek when regulating emotions, the research of Dr. Maya Tamir stands out. She considers that emotional goals determine the destination and regulatory strategies and are the cognitive representation of the desired emotional state (Tamir, 2016). Other research focuses on addressing personal variables such as attachment and temperament that explain individual differences in the development of emotional regulation.

Zimmerman stands out among the authors who investigate the relationship between emotional regulation and attachment. This study perspective assumes the recognition of the existence of regulation patterns acquired from infancy according to the bond established with attachment figures. In terms of Bowlby, an internal working model that influences not only regulation but also the genesis of emotion. Initially, the baby lacks the capacity to regulate itself; it's others, especially attachment figures, who regulate it (Spangler et al., 1994). Attachment influences emotional regulation in all stages of life due to the internal working model generated in attachment care experiences (Bowlby, 1969; Bretherton and Munholland, 2008; Zimmerman, 1999). These internal working models include emotional and motivational characteristics, cognitive knowledge, and scripts of when and how to display attachment behavior, the availability of attachment figures, reactions to distress, or self-regard as worthy of love or not (Spangler and Zimmermann, 2014). Therefore, attachment influences behavioral organization and emotional regulation (Bowlby, 1980; Cassidy, 1994; Zimmermann et al., 2001).

Eisenberg et al. (2010) focus on differences in emotion management according to temperamental characteristics, which largely explain more automatic emotional reactivity (Bargh and Williams, 2007). These automatic, unconscious, and uncontrolled responses to stimuli or cognitions relevant to emotions can sometimes be more reliable and effective than more conscious responses (Bargh and Williams, 2007). Although inheritance can predispose us to certain types of behavior or reactions, environmental factors that mediate or moderate that relationship could be changed. During the upbringing period, both children's and parents' behavior can be modified, which is important because the mutual influence of both mediates temperamental manifestation (Eisenberg et al., 2010).

Any review of the emotional regulation construct is complex due to the vast volume of research developed over decades, which makes the present synthesis undoubtedly limited and insufficient, but it highlights a fact: there are many aspects and dimensions involved in emotional regulation, which perhaps signals the need to use a broader, dimensional construct that can give rise to different explanatory models of interaction between variables, thus allowing for a better understanding of the phenomenon.

## 3 Emotional intelligence and emotional competence

Emotional regulation has also been studied from the perspective of Salovey and Mayer's Theory of Emotional Intelligence (Mayer and Salovey, 1997). This model argues that Emotional Intelligence (EI) consists of four components: emotional perception and expression, emotional facilitation, emotional understanding, and emotional regulation. They consider emotional regulation as a manifestation of EI and theorize that higher EI leads to greater emotional regulation skills. Furthermore, they argue that emotional regulation is based on individuals' experiences, culture, and personal needs. They define emotional regulation as the capacity to be open to both positive and negative emotional states, reflect on them to determine if the accompanying information is useful without repressing or exaggerating them, and regulate our own emotions and those of others.

They categorized the construct as a type of intelligence because, according to their studies, it meets the criteria to be considered as such (Mayer et al., 2000). Without going into detail on this aspect, as it would go beyond the scope of the present study, it is worth briefly mentioning the three criteria that, according to the cited study, Emotional Intelligence meets to support that it is a type of intelligence: (i) conceptual, (ii) correlational, and (iii) developmental. (i) We say that Emotional Intelligence meets the conceptual criterion because, like other types of intelligence, it reflects intellectual performance by individuals. This is manifested in skills that can be measured. (ii) The correlational criterion refers to the fact that the skills described by Emotional Intelligence (like other types of intelligence) are related to each other because they are similar, but they are also distinguished from each other, with each being different from the others. (iii) The third criterion it meets is that intelligence develops with age and experience, from childhood to adulthood.

From the systematization of the construct by these authors, three lines of development have emerged: (i) the aforementioned model by Mayer and Salovey, which is based on ability; (ii) the so-called “mixed model” by Bar-On (2000). This model is based on social-emotional intelligence and is a kind of interrelationship between emotional and social competencies, facilitators, and skills that determine how efficient individuals are in understanding and expressing themselves, understanding others, relating to others, and facing challenges in this regard; (iii) Goleman's (1996) model. This model focuses mainly on competencies applied to success in the workplace. It maintains that emotional intelligence is based on five elements: knowing one's own emotions, regulating one's own emotions, self-motivation, recognizing emotions in oneself and others, and managing relationships (Fernández-Berrocal and Extremera, 2006; Gómez Leal et al., 2018). Goleman's model was widely disseminated, as it was publicized in mass media and received widespread public reception.

The repeated criticism found in various articles focuses on the confusion of the construct with a wide variety of terms that actually define personality traits (such as enthusiasm, zeal, empathy, general character) (Mayer et al., 2008, 2011).

Emotional regulation is also considered as one of the competencies within some emotional competence models. For the development of this construct, we will rely on Carolyn Saarni's definition, who was the first author to develop the concept (Fragoso-Luzuriaga, 2015; Mayer and Salovey, 1997).

Carolyn Saarni, a constructivist psychologist who has focused her research on early childhood education, defines emotional competence as “the demonstration of self-efficacy to control emotional reactions in social interactions.” Based on this definition, the author presents a model that explores the factors and skills that contribute to the development of a mature emotional response, based on achieving an individual's social goals. The factors contributing to the development of emotional competence are self-identity development, moral awareness of what is right and wrong in certain social contexts, with their own codes, and personal (developmental) history (Saarni, 1997, 1999).

As a summary in explaining the construct, it is worth mentioning the eight skills that the author highlights as characteristics of emotionally competent individuals. The skills highlighted by the author are (a) awareness of one's own emotional state, (b) ability to discern emotions in others, (c) ability to describe one's own emotions, (d) empathy, (e) ability to distinguish between internal emotions and the externalization or manifestation of those emotions, (f) adaptive capacity to deal with adverse emotions that generate stress, (g) awareness of the role of emotions in relationship structure, (h) ability to be emotionally effective.

This construct, as defined by the author, refers to a process of individual maturation through which individuals develop skills to ensure that their emotions do not negatively interfere with their performance in social relationships.

Carolyn Saarni, in her work developing the emotional competence construct, makes a distinction between it and emotional intelligence, taking the definition of Mayer and Salovey (1997) for this purpose.

The cited study points out that the definition of emotional intelligence does not refer to the ethical values surrounding the individual or to the development of self-identity. The author emphasizes that in the development of these constructs, insufficient attention has been paid to the role played by context, environment, and self-development in an individual's emotional functioning (Saarni, 1999).

In a more recent work, the same author points out in more detail the three fundamental differences between the two constructs: (i) Emotional competence is a set of acquired skills that develop interdependently. The Emotional Intelligence model does not propose skill development; (ii) individuals who are emotionally competent react to environmental emotional stress with skills, while emotionally intelligent people respond through traits that are inherent to their personality; (iii) emotional competence emphasizes personal integrity as a factor contributing to emotionally competent and mature functioning. The moral aspect as a component of emotional competence has no parallel in the conceptualization of emotional intelligence (Saarni, 2011).

As we have seen, when addressing the question “how to intervene in emotional experience?” there are various concepts, explanations, and meanings. The challenge that this situation may pose is to find a way to integrate the diversity of contributions in order to gain a deeper understanding of the complexity of emotional experience. Reviving the term “affectivity” in the field of research and theoretical development in psychology is the option proposed in this study.

## 4 The importance of recovering the term affectivity in psychology

The study of emotions spans multiple disciplines, including psychology, neurology, and ethics. However, a comprehensive understanding of emotions can only be achieved through anthropology (Malo, 2004). Anthropology views emotion as an inherently affective experience. Affectivity is the human faculty that enables us to experience emotions, feelings, affections, and moods. It is our capacity to be affected, engaged, and called upon by our surroundings, allowing us to respond accordingly. This engagement with reality is possible because some aspects of it resonate with us, prompting interaction (Domínguez, 2007).

### 4.1 Affectivity as a fundamental human faculty

Affectivity is crucial for our interaction with reality, guiding our attention, consciousness, and will. Without this faculty, we would remain indifferent to the multitude of stimuli we encounter. It is this selective responsiveness that initiates our engagement with these stimuli. Thus, affectivity can be seen as an initial characteristic that disposes us to reality, propelling us into action. This process occurs not only in response to concrete realities but also to imaginary ones, such as memories, aspirations, and dreams (Domínguez, 2007).

### 4.2 Distinguishing affectivity from emotion

Research shows that the most studied aspect of affectivity is emotion, leading to the frequent misconception of using the terms affectivity and emotion interchangeably (Surallés, 2009; Sancho and Martínez, 2011). However, affectivity encompasses more than just emotions; it is the broader capacity to be influenced by the meaningful aspects of reality (Costa-Lobo et al., 2020; Souza et al., 2013). This implies a combination of physical, cognitive, behavioral, and sociocultural mechanisms that include moods, emotions, and affections (Hervás and Vázquez, 2006).

### 4.3 Historical perspective on emotion

The term “emotion” originates from the Latin word “emovere,” meaning to induce movement. It was popularized in psychological discourse through the works of Hume (Treatise of Human Nature) and Smith (The Theory of Moral Sentiments), and later by Darwin, who viewed emotion as a reactive, primarily physiological experience with significant similarities to animal experiences (Sacristán, 2020). Modern psychology, particularly from the 1960s onwards, adopted this Darwinian perspective, focusing on the external expression of emotions rather than their internal experience. This shift led to a mechanistic view of affectivity, reducing it to bodily expressions rather than aspects of the soul, as seen in earlier theories by James-Lange and Cannon-Bard.

### 4.4 The shift from emotion to affectivity

Dixon (2003) illustrates how the 19th century saw the rise of emotions as a distinct psychological category, replacing older concepts such as appetites, passions, feelings, and affections. By comparing medieval theological psychologies and those of the 18th century with Darwin and William James, Dixon argues that the dominance of a single descriptive category (emotion) is inadequate. Focusing exclusively on emotion fails to capture the variety of affective experiences. Restoring the concept of affectivity allows for a more comprehensive and integrative approach to human experience.

### 4.5 Integrating affectivity into psychological study

Within the psychology of emotion, other affective experiences like feelings and affections are acknowledged but lack the same theoretical and research development. Feelings, originating from romanticism, are seen as lasting impressions left by encounters. Affections express preferences for people or things, playing a defining and explanatory role in our lives (Sacristán, 2022).

By focusing on the psychology of emotion rather than affectivity, we miss the opportunity to study all affective experiences and their interactions. Limiting our focus to emotions means neglecting the more enduring and structuring aspects of affectivity, such as affections and feelings, and concentrating only on the immediate, reactive aspect of emotions.

There is a noticeable gap in psychological studies addressing emotions or other affective phenomena from a model of affectivity. This gap hinders our understanding of the genesis and interrelation of affective experiences with other dimensions of the person, such as cognition and will. Typically, the term “affective” is used to refer to positive or negative affective experiences (Watson et al., 1988), but a more thorough approach would encompass the full spectrum of affective experiences and their significance. Therefore, recovering and emphasizing the term affectivity in psychological discourse is essential for a holistic understanding of human experience.

Russell's (2003, 2005, 2009) proposal, with significant theoretical and research development, highlights the insufficiency of various emotion theories and advocates for the search for broader explanatory models capable of integrating and explaining the relationship between emotional phenomena that have been studied so far. Specifically, Russell's proposal focuses on a core affect, which structures the individual and explains the psychological processes that arise from interaction with reality, leading to subjective affective experience.

While it is a novel proposal that opens new and interesting paths, it does not resolve a fundamental issue: the biological and psychological dimensions, as well as their integration, are not sufficient to fully understand the affective experience. Why does a reality affect me the way it does? Answering this question requires a necessary reference to the affected self, to its way of valuing reality. It is not enough to understand how its value systems have been formed; it is also crucial to consider how they are managed, developed, and enriched by the conscious self.

When we try to understand affective phenomena, due to their subjective, personal, and evaluative nature, it is essential to address issues such as the self, identity, consciousness, freedom, ideals, and the meaning of life. Thus, the possibility arises that a spiritual dimension may ultimately explain the integration of affective phenomena, emphasizing the importance of the ideal of life (Arnold, 1960).

## 5 Conclusions

After reviewing other constructs related to the affective dimension of the individual that have a notable presence in scientific literature, we can conclude that most of them primarily focus on emotional experience, without paying attention to other affective experiences. This may be because emotion is more easily observable and measurable within the complexity of human affect. However, this provides us with a limited and partial view of the human experience, as emotion is just one reaction.

The studied constructs delve into the capacities or abilities that enable better management of these emotional reactions. While Saarni's model of emotional competence relates these skills to other dimensions of the individual, such as moral awareness, identity, and personal history, and studies by Zimmerman on attachment and emotional regulation, as well as authors studying the influence of temperament and emotional regulation, link emotional management to all dimensions of the individual and their development. However, these constructs overlook the importance of affection, such as the bond or preference of the individual, which plays a fundamental role in structuring the individual's being and, therefore, in their emotional reactions.

We can therefore conclude that there is a need for a model that encompasses both emotion and mood, as well as affection, and defines the structure and relationship between them. In summary, an affective psychological model is required to capture the complexity of the human experience in its entirety.

A more comprehensive model would guide clinical and educational interventions that foster a deeper understanding of the human experience, as well as the processes of healing and growth. This approach would lead to a more integrative practice, avoiding reductionism or limitations. We would not focus solely on reactive emotional events and their management, but rather seek to better understand why they occur in the person, addressing their root causes. It would involve moving beyond reaction and control, to examine the origin, the cause, and potential interventions.

From a research perspective, this model raises longstanding and significant questions in psychology, related to the idea of a self that integrates personal experience, which originates from how reality affects the individual. Ultimately, this necessitates an anthropological reflection on the structure of the human being and the importance of a spiritual dimension, as a conscious and free subject, to fully explain all human behavior, which begins with an affective experience, in the way one is affected by something.

## References

[B1] Adrian M. Zeman J. Veits G. (2011). Methodological implications of the affect revolution: a 35- year review of emotion regulation assessment in children. J. Exp. Child Psychol. 110, 171–197. 10.1016/j.jecp.2011.03.009 21514596

[B2] Arnold M. (1960). Emotion and Personality. Volume I: Psychological Aspects. Volume II: Neurological and Physiological Aspects. New York, NY: Columbia University Press.

[B3] Bargh J. A. Williams L. E. (2007). “The nonconscious regulation of emotion,” in Handbook of Emotion Regulation, ed. J. J. Gross (New York, NY: The Guilford Press), 429–445.

[B4] Bar-On R. (2000). “Emotional and social intelligence: insights from the emotional quotient inventory,” in The Handbook of Emotional Intelligence, eds. R. Bar-On, and J. D. A. Parker (New York, NY: Jossey-Bass), 363–388.

[B5] Bowlby J. (1969). Attachment (Vol 1). Attachment and Loss. New York, NY: Basic Books.

[B6] Bowlby J. (1980). Loss, Sadness and Depression (Vol 3). Attachment and Loss. New York, NY: Basic Books.

[B7] Bretherton I. Munholland K. A. (2008). “Internal working models in attachment relationships: elaborating a central construct in attachment theory,” in Handbook of Attachment: Theory, Research, and Clinical Applications, 2nd Edn, eds. J. Cassidy, and P. R. Shaver (New York, NY: The Guilford Press), 102–127.

[B8] Cassidy J. (1994). Emotion regulation: Influences of attachment relationships. Monogr. Soc. Res. Child Dev. 59, 228–283. 10.1111/j.1540-5834.1994.tb01287.x 7984163

[B9] Cole P. Martiin S. Dennis T. (2004). Emotion regulation as a scientific construct: methodological challenges and directions for child development research. Child Dev. 75, 317–333. 10.1111/j.1467-8624.2004.00673.x 15056186

[B10] Costa-Lobo C. Matamá J. Nascimento D. (2020). Reasoning, affectivity and school performance at the end of Portuguese elementary education: which variables to consider? Paidéia 30:e3008. 10.1590/1982-4327e3008

[B11] Davidson R. J. (1994). “Un emotion, mood, and related affective constructs,” in The Nature of Emotion: Fundamental Questions, eds. P. Ekman, and R. J. Davidson (Oxford: Oxford University Press), 51–55.

[B12] Dixon T. (2003). From Passion to Emotions: The Creation of a Secular Psychological. Cambridge.

[B13] Domínguez X. M. (2007). De todo corazón. Fundación Emmanuel Mounier

[B14] Duckworth K. Rodie A. Alice M. Emma S. John V. (2009). Self-Regulated Learning: A Literature Review. Wider Benefits of Learning Research Report, 33. Available at: https://discovery.ucl.ac.uk/id/eprint/10015842

[B15] Eisenberg N. Spinrad T. Eggum N. (2010). Emotion-related self-regulation and its relation to children's maladjustment. Annu. Rev. Clin. Psychol. 6, 495–525. 10.1146/annurev.clinpsy.121208.131208 20192797 PMC3018741

[B16] Ekman P. (1994). “All emotion are basi,” in The Nature of Emotion: Fundamental Questions, eds. P. Ekman, and R. J. Davidson (Oxford: Oxford University Press), 15–19.

[B17] Fernández-Berrocal P. Extremera N. (2006). La investigación de la Inteligencia Emocional en España. Ansiedad y Estrés 12, 139–153.

[B18] Fragoso-Luzuriaga R. (2015). Inteligencia emocional y competencias emocionales en educación superior, ¿un mismo concepto? *Rev. Iberoamericana Educ. Super*. 10.22201/iisue.20072872e.2015.16.154

[B19] Goldsmith H.H. (1994). “Parsing the emotional domain from a developmental perspective,” in The Nature of Emotion: Fundamental Questions, eds. P. Ekman, and R. J. Davidson (Oxford: Oxford University Press), 68–73.

[B20] Goleman D. (1996). Inteligencia Emocional. New York, NY: Kairós.

[B21] Gómez Leal R. Gutierrez Cobo M. J. Cabello R. Fernández Berrocal P. (2018). The relationship between the three models of emotional intelligence and psychopathy: a systematic review. Front. Psychiatry 9:307. 10.3389/fpsyt.2018.00307 30050475 PMC6052135

[B22] Gross J. J. (1999). Emotion, regulation: past, present, future. Cognit. Emot. 13, 551–573. 10.1080/026999399379186

[B23] Gross J. J. (2014). Handbook of Emotion Regulation. New York, NY: The Guilford.

[B24] Gross J. J. Thompson R. A. (2007). “Emotion regulation: conceptual foundations,” in Handbook of Emotion Regulation, ed. J. J. Gross (New York, NY: The Guilford Press), 3–24.

[B25] Hervás G. Vázquez C. (2006). La regulación afectiva: Modelos, investigación e implicaciones para la salud mental y física. Rev. Psicol.Gen. Apl. 59, 9–36.

[B26] Malo A. (2004). Antropologí*a de la Afectividad*. New York, NY: Eunsa.

[B27] Martínez C. (2018). ¿*Qué caracteriza la madurez emocional y de la personalidad? Quiénes somos, cuestiones en torno al ser humano*. New York, NY: Eunsa.

[B28] Mayer J. D. Salovey P. (1997). “What is emotional intelligence?” in Emotional Development and Emotional Intelligence: Educational Implications, eds. P. Salovey, and D. J. Sluyter (New York, NY: Basic Books), 3–34.

[B29] Mayer J. D. Salovey P. Caruso D. (2000). “Models of emotional intelligence,” in Handbook of Intelligence, ed. R. Sternberg (Cambridge: Cambridge University Press), 396–420.

[B30] Mayer J. D. Salovey P. Caruso D. R. (2008). Emotional intelligence: new ability or eclectic traits? Am. Psychol. 63, 503–517. 10.1037/0003-066X.63.6.503 18793038

[B31] Mayer J. D. Salovey P. Caruso D. R. Cherkasskiy L. (2011). “Emotional intelligence,” in The Cambridge Handbook of Intelligence, eds. J. Sternberg and S. B. Kaufman (Cambridge: Cambridge University Press), 528–549.

[B32] Reeve J. (2009). Motivación y Emoción. New York, NY: McGrawHill.

[B33] Russell J. A. (2003). Core affect and the psychological construction of emotion. Psychol. Rev. 110, 145–172. 10.1037/0033-295X.110.1.145 12529060

[B34] Russell J. A. (2005). Emotion in human consciousness is built on core affect. J. Conscious. Stud. 12, 26–42.21241309

[B35] Russell J. A. (2009). Emotion, core affect, and psychological construction. Cogn. Emot. 23, 1259–1283. 10.1080/02699930902809375

[B36] Saarni C. (1997). “Emotional competence and self-regulation in childhood,” in Emotional Development and Emotional Intelligence: Educational Implications, eds. P. Salovey, and D. J. Sluyter (New York, NY: Basic Books), 35–69.

[B37] Saarni C. (1999). The Development of Emotional Competence. New York, NY: Guilford Press.

[B38] Saarni C. (2011) “Emotional competence and effective negotiation: the integration of emotion understanding, regulation, and communication,” in Psychological and Political Strategies for Peace Negotiation, eds. F. Aguilar, and M. Gallucio (New York, NY: Springer), 55–74..

[B39] Sacristán R. (2020). Movidos por el amor. Estudio del dinamismo afectivo. San Dámaso.

[B40] Sacristán R. (2022). Educación y afectividad. Jalones para una pedagogía del amor. Didaskalos.

[B41] Sancho D. Martínez A. (2011). Afectividad positiva y salud. Enfermería Global 10, 120–124. 10.4321/S1695-61412011000400010

[B42] Souza V. L. T. Petroni A. P. Andrada P. C. (2013). Affectivity as an aspect of teachers' identity constitution: a view from psychology. Psicol. Soc. 25, 527–537. 10.1590/S0102-71822013000300007

[B43] Spangler G. Schieche M. Ilg U. Maier U. Ackermann C. (1994). Maternal sensitivity as an external organizer for biobehavioralregulation in infancy. Dev. Psychobiol. 27, 425–437. 10.1002/dev.420270702 7843497

[B44] Spangler G. Zimmermann P. (2014). Emotional and adrenocortical regulation in early adolescence: prediction by attachment security and disorganization in infancy. Int. J. Behav. Dev. 38, 1–13. 10.1177/0165025414520808

[B45] Surallés A. (2009). De la intensidad o los derechos del cuerpo. La afectividad como objeto y como método. Runa XXX, 29–44.

[B46] Tamir M. (2016). Why do people regulate their emotions? A taxonomy of motives in emotion regulation. Person. Soc. Psychol. Rev. 20, 199–222. 10.1177/1088868315586325 26015392

[B47] Thompson R. A. (1994). “Emotion regulation: a theme in search of definition,” in The Development of Emotion Regulation and Dysregulation: Biological and Behavioral Aspects. Monographs of the Society for Research in Child Development 59 (2–3, Serial no. 240), ed. N. A. Fox, 25–52. 7984164

[B48] Watson D. Clark L. A. Tellegen A. (1988). Development and validation of brief measures of positive and negative affect: the PANAS scales. J. Pers. Soc. Psychol. 54, 1063–1070. 10.1037/0022-3514.54.6.1063 3397865

[B49] Zimmerman P. (1999). Structure and functions of internal working models of attachment and their role for emotion regulation. Attach. Hum. Dev. 1, 291–306. 10.1080/14616739900134161 11708228

[B50] Zimmermann P. Maier M. A. Winter M. Grossmann K. E. (2001). Attachment and adolescents' emotion regulation during a joint problem-solving task with a friend. Int. J. Behav. Dev. 25, 331–343. 10.1080/01650250143000157

